# Chromosomally Integrated Human Herpesvirus 6: Models of Viral Genome Release from the Telomere and Impacts on Human Health

**DOI:** 10.3390/v9070184

**Published:** 2017-07-12

**Authors:** Michael L. Wood, Nicola J. Royle

**Affiliations:** Department of Genetics, University of Leicester, University Road, Leicester LE1 7RH, UK; mlw25@leicester.ac.uk

**Keywords:** human herpesvirus 6, HHV-6, telomere, viral reactivation, replication, integration

## Abstract

Human herpesvirus 6A and 6B, alongside some other herpesviruses, have the striking capacity to integrate into telomeres, the terminal repeated regions of chromosomes. The chromosomally integrated forms, ciHHV-6A and ciHHV-6B, are proposed to be a state of latency and it has been shown that they can both be inherited if integration occurs in the germ line. The first step in full viral reactivation must be the release of the integrated viral genome from the telomere and here we propose various models of this release involving transcription of the viral genome, replication fork collapse, and t-circle mediated release. In this review, we also discuss the relationship between ciHHV-6 and the telomere carrying the insertion, particularly how the presence and subsequent partial or complete release of the ciHHV-6 genome may affect telomere dynamics and the risk of disease.

## 1. Introduction

**Figure 1:**
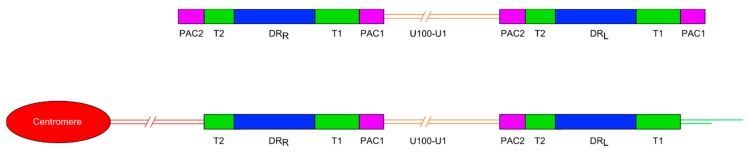
Diagram showing the general organisation of the HHV-6A and HHV-6B genomes. Upper panel shows the organisation of the non-integrated linear DNA genome of HHV-6A or -6B. The lower panel shows the loss of the terminal packaging sequences 1 and 2 (PAC1 and PAC2) following chromosomal integration in a telomere. DR_L_ and DR_R_: Direct repeat regions; T1 and T2: Variable length telomere-like repeat regions.

Human herpesvirus 6A and human herpesvirus 6B (HHV-6A and HHV-6B) are two members of the *Betaherpesvirinae* subfamily of herpesviruses [1]. Multiple studies have shown that chromosomally integrated HHV-6 (ciHHV-6) is usually found in a single telomere in humans but this integration may occur at a variety of sites, including 17p13.3, 22q13.3, 1q44, 9q34.3, 10q26.3, 11p15.5, 19q13.4, 18p11.3, 18q23, 7q22, 6q and Xp [2,3,4,5,6,7,8,9,10,11,12,13,14,15]. The HHV-6A and -6B linear double-stranded DNA genomes comprise a unique region of 143–145 kb, flanked by two identical direct repeat regions (DR_L_ and DR_R_, each approximately 8 kb) which are in turn flanked by variable length telomere-like repeat regions (T1 and T2) and packaging sequences (PAC1 and PAC2) (Figure 1). These viral genomes contain 86 genes. The T1 region is variable in length between viral isolates and comprises TTAGGG repeats interspersed with degenerate telomere-like repeats. The T2 region is shorter than T1 and comprises a perfect array of (TTAGGG)_n_ that can also vary in length between isolates [16,17,18]. The T2 region is essential for viral integration as in vitro recombinant viruses lacking T2 had a very low integration efficiency whereas deletion of T1 had only a very small effect on integration efficiency [19]. This is consistent with the suggestion that homology-dependent recombination between T2 and the human telomere, facilitated by cellular factors or the products of viral genes, is the first step in HHV-6 chromosomal integration [11]. The HHV-6 U94 gene appeared to be a good candidate for promoting integration, being a putative integrase replete with DNA-binding, exonuclease and helicase activity [20]. However, recently it has been shown that U94 is dispensable for integration [21], suggesting that there may be several independent integration mechanisms.

### 1.1. Organisation of ciHHV-6 Genome

Most ciHHV-6 genomes that have been characterised have a 2:1 ratio of DR to unique regions, consistent with a single integration of a full length HHV-6 genome (Figure 1). More recently, the use of digital-droplet PCR has confirmed that most ciHHV-6+ individuals have only a single, full-length integration [22,23]. To date, the ciHHV-6 genome has always been found in the same orientation, with DR_R_ at the centromere-proximal end and DR_L_ towards the distal end of the chromosome. Integration via homology-dependent recombination with telomeric DNA results in loss of the terminal DR_R_-PAC2 sequence [11]. The terminal PAC1 sequence at the distal end of DR_L_ is also absent from ciHHV-6 genomes and the T1 region becomes part of the telomere at the end of the viral genome [24]. It is unknown whether PAC1 is lost as a result of recombination during the integration process or because of gradual erosion prior to addition of telomere repeats by telomerase. Other organisations of integrated HHV-6 genomes have also been identified including arrangements with three DRs and one unique region [14] or just a single DR without the unique region in the telomere [24] and possibly in non-telomeric positions [25].

### 1.2. Germline Versus Somatic Integration and HHV-6 Latency

Vertical transmission of ciHHV-6 is possible once the viral genome has integrated into germline cells, resulting in the descendants inheriting ciHHV-6 in a Mendelian manner. Those with inherited ciHHV-6 carry an integrated copy of the virus in every nucleated cell. The proportion of inherited ciHHV-6 individuals varies from 0.2% in Japan [7] to approximately 2% in Western populations depending on the population sampled [26,27,28,29,30] but it is estimated to be around 1% globally. Although HHV-6 has the capacity for telomeric integration, the germline integration rate has not been investigated. Factors that are expected to influence this rate include the likelihood of the viral genome or packaged virus migrating to the gonads and in gaining access to gametes, or perhaps early meiotic cells where telomeric integration by recombination may be more likely. A variety of herpesviruses, including HHV-6, can be detected in semen samples from healthy donors; HHV-6 was found in 13.5% of Danish semen samples [31] and at higher frequency in men attending an infertility clinic in Greece [32]. However, the frequency of de novo telomeric integration in germ cells remains unknown. 

Although approximately 1% of the global population inherit ciHHV-6, greater than 90% of the global population is infected by HHV-6B in early infancy owing to horizontal transmission of viral particles shed in saliva. The prevalence of primary infections by HHV-6A is considerably lower than by HHV-6B in European, North American and Japanese populations studied [33,34,35]. In contrast, HHV-6A appears to be the dominant form of primary infection in some African populations [36]. Recently, it was demonstrated using digital droplet PCR that coinfection of HHV-6A and HHV-6B may be as high as 50% [37]. Following initial infection, HHV-6 becomes latent in a small proportion of somatic cells [38,39,40,41,42]. To date, episomal forms of HHV-6 have not been detected in latency and this, with in vitro experiments, suggests that latency is achieved by chromosomal integration in somatic cells [11]. Assuming somatic ciHHV-6 is indeed the latent state, viral reactivation requires the entire HHV-6 genome to be released or copied from the telomere for the production of viral particles.

Once the HHV-6 genome has undergone germline or somatic integration, it will be maintained by the human replication and DNA repair pathways and so the mutation rate across the viral genome is predicted to drop to that of the human genome. This is likely to create differences in the rate of evolution between integrated and non-integrated HHV-6 genomes. Continued passage of inherited ciHHV-6 through many human generations could then impact on the evolution of the viral genome and this is now beginning to be explored. 

### 1.3. Telomeres, Length Regulation and the Effect of ciHHV-6

Telomeres protect chromosomes by ensuring that chromosome ends are not mistakenly identified as double-strand-breaks by DNA damage response systems as such errors can lead to inappropriate repair by non-homologous end joining or homology dependent recombination mechanisms [43,44,45]. In addition, telomeres provide a mechanism for managing the inevitable erosion of DNA sequences that occurs during lagging strand synthesis of linear DNA molecules, combined with end-processing that generates the essential 3’ (TTAGGG)_n_ single strand overhang of telomeres. Most essential functions of telomeres are achieved or regulated by the six protein structure called Shelterin [46] and the enzyme telomerase, the reverse transcriptase that adds telomere repeats onto the G-rich strand of the telomere [47]. Shelterin binds to double stranded (TTAGGG)_n_ via the Telomeric Repeat Binding Factors 1 snd 2 (TRF1 and TRF2) components and to the single-strand overhang via Protection of Telomeres 1 (POT1). These Shelterin components, with their various interacting partners (TIN2, RAP1 and TPP1), orchestrate activities that affect telomeric DNA [48]. This includes preventing non homologous end joining and homology dependent recombination repair at telomeres, and regulating access to the single strand overhang by telomerase. In human somatic cells, telomeres play a significant role in cancer avoidance [49]. Telomerase is tightly regulated in human tissues and it is normally only active in some stem cells and in the germline. Consequently, successive rounds of replication result in the gradual shortening of telomeres in somatic cells; this eventually precipitates a DNA damage response and the onset of cellular senescence. 

The presence of ciHHV-6 within a telomere could disturb some essential features of telomeres, for example because the viral genome contains potential binding sites for the Shelterin complex. Analysis of lymphoblastoid cell lines from inherited ciHHV-6 carriers has shown that the telomere on the end of the integrated viral DR_L_-T1 is eroded at a similar rate to other telomeres, suggesting no major defect in replication or single strand end-processing [24]. Measurement of individual telomeres in sperm DNA from four ciHHV-6 carriers showed that ciHHV-6 associated telomeres were long, indicating they can be lengthened and maintained by telomerase. In contrast, in lymphoblastoid cell lines, the median length of the ciHHV-6 associated telomere was often short compared to other telomeres measured [24]. Further work is needed to understand fully the dynamic processes that may have a differential effect on a ciHHV-6 associated telomere compared to others within the same cell.

## 2. ciHHV-6 and Disease Risk

There are several ways in which inherited or somatic ciHHV-6 could affect human health, for example: (i) disturbance of telomere function due to a large, predominantly non-telomeric, insertion; (ii) the consequences of viral gene expression from the ciHHV-6 genome; (iii) partial or complete loss of the HHV-6 genome from the telomere and (iv) full viral reactivation. The incidence of inherited ciHHV-6 was found to be higher in hospitalised individuals than in the general population [27,50], possibly suggesting an impact on health, although not associated with any particular disease. More recently, an extensive study has shown that carriers of inherited ciHHV-6 in Canada have a higher risk of developing angina pectoris [30].

### 2.1. Reactivation of Inherited ciHHV-6

Currently, there are a small number of studies that have demonstrated full viral reactivation in carriers of inherited ciHHV-6. These include a series of studies that show reactivation of inherited ciHHV-6A and ciHHV-6B in mothers resulting in transplacental infection of their non-ciHHV-6 babies [26,28,51]. Reactivation of inherited ciHHV-6 has also been convincingly demonstrated in a boy with X-linked severe combined immunodeficiency (X-SCID) arising from a maternally inherited mutated *IL2RG* gene. The child inherited ciHHV-6A in chromosome 22 from his father and this was confirmed to have reactivated by sequence analysis. Reactivation was believed to be due to the compromised immune system of the child who developed haemophagocytic syndrome. The patient became asymptomatic after antiviral treatment and haematopoietic stem cell transplant were successful in reducing the level of HHV-6 DNA in blood titres to zero (Endo et al., 2014). Kühl et al (2015) have investigated reactivation of inherited ciHHV-6 in myocarditis patients. They found that the inherited ciHHV-6 frequency was slightly, but not significantly, higher in this patient cohort (1.1%) compared to the wider, healthy population. The cardiac symptoms were rapidly alleviated by anti-viral treatment and could reoccur when treatment was withdrawn. The authors proposed that inherited ciHHV-6 can replicate in degenerating cardiomyocytes and may lead to cardiac symptoms.

### 2.2. Somatic HHV-6 Reactivation and Encephalitis

Reactivation of ciHHV-6 is a common occurrence in recipients of organ transplantation [52]. Numerous studies, predominantly involving transplant centres in Japan, have described an association between full reactivation of latent HHV-6 and encephalitis in recipients of hematopoietic cell transplantation (HCT) [53,54,55,56]. One study [57] monitored HHV-6 reactivation and incidence of encephalitis in recipients for 70 days following HCT. A high level of HHV-6 reactivation occurred in 86 of 230 HCT recipients (37.0%) and of these 7 (8.1%) developed encephalitis whereas no individuals with low level or no reactivation developed encephalitis. In this clinical scenario, immunosuppression in the HCT recipients is believed to have led to somatic HHV-6 reactivation. However, several studies [58,59,60,61,62], while in agreement that HCT can lead to HHV-6 reactivation, do not find an association between reactivation and encephalitis. Perhaps notably, these studies did not involve patients at Japanese transplant centres. Furthermore, an association between HHV-6 reactivation and encephalitis has been demonstrated in a North American cohort of bone marrow transplant recipients [63]. These findings, among others, paint a somewhat puzzling picture of the relationship between HHV-6 reactivation and encephalitis, suggesting that while transplantation often leads to virus reactivation, this tends not to lead to serious disease.

### 2.3. ciHHV-6 Loss, Telomere Instability and Disease

Primary-effusion lymphoma (PEL) B-cell malignancy usually occurs in human immunodeficiency virus-positive (HIV^+^) individuals with latent HHV-8 but can also occur in HIV^−^ individuals with no detectable HHV-8 [64,65]. The name HHV-8-unrelated PEL-like lymphoma (full name: HHV-8-unrelated primary-effusion lymphoma-like lymphoma) may be misleading as these malignancies may more closely resemble other lymphomas or could be distinct rare diseases. Regardless, we have described one such HHV-8-unrelated PEL-like lymphoma in an elderly woman (HIV^−^, lacking detectable HHV-8 and without a compromised immune system) who was also a carrier of inherited ciHHV-6A integrated in a 19q telomere. Interestingly, the inherited ciHHV-6A genome was absent from the lymphoma cells. The exact mechanism underlying the loss of the ciHHV-6 genome is not known, but the lymphoma cells retained both copies of chromosome 19 and several lines of evidence indicate it was lost through a specific clonal event during or prior to the initiation of the HHV-8-unrelated PEL-like lymphoma. 

The inherited ciHHV-6A in the woman’s normal cells and in her two brothers, who carried the same 19q ciHHV-6A, were identical, containing a full set of HHV-6A genes and without any gross rearrangements. It is tempting to speculate that loss of inherited ciHHV-6A genome was causative in the lymphomagenesis in this woman, but it was not possible to discriminate between this hypothesis and the contrary possibility that loss of the inherited ciHHV-6 genome was coincidental but unrelated to the lymphoma development. 

Recent studies have demonstrated that chromosome looping can bring the telomere into proximity of genes up to 10 Mb away but that the most distant interactions do not occur when the telomere is short [66]. The authors suggest that expression of sub-telomeric genes, for example human telomerase reverse transcriptase (hTERT) [67], may be altered by changes in telomere length due to telomere position effect over long distances (TPE-OLD). Integration of a 160 kb HHV-6 genome into a telomere may affect expression of human genes by interrupting TPE-OLD. It may also be important to consider the effect that telomere length could have on viral gene expression through long-range interactions. Changes in expression of human or viral genes may lead to a predisposition to disease, or release of the viral genome. Given the antiquity of some inherited ciHHV-6 integration events that we have found, co-evolution of the human and viral genomes may have compensated for any initial disruption but the sudden loss of the ciHHV-6 genome could once again disrupt the equilibrium and telomere position effects could alter gene expression.

## 3. Reactivation of Inherited or Somatic ciHHV-6

The first step in full viral reactivation from ciHHV-6 is release of the viral genome, either through excision from the telomere or by being copied entirely from its location in the chromosome. Here, we propose possible models for viral genome release.

### 3.1. Transcription and Reverse Transcription of ciHHV-6

Telomere repeat-containing RNA (TERRA) is produced by the transcription of telomeric DNA, with transcription initiation starting upstream of the telomere [68]. Several studies have highlighted the complex role that TERRA plays in regulating telomere length when either telomerase [69] or the alternative lengthening of telomeres (ALT) maintenance mechanisms are active [70,71] alternatively by accelerating the shortening of telomeres when no telomere maintenance mechanism is active [72]. TERRA transcripts of up to 9 kb, have been reported, which may reflect the average length of the telomeres. As considerably longer non-coding RNAs are produced from other regions of the human genome, a hypothetical first step of ciHHV-6 reactivation could involve transcription of the entire ciHHV-6 genome from a TERRA transcriptional start site. Again hypothetically, the long RNA could be converted to a DNA:RNA hybrid by a reverse transcriptase, for example the open reading frame 2 (ORF2) of the human long interspersed nuclear element 1 (L1). In the somewhat unlikely event that double-stranded HHV-6 could be generated by such a mechanism, the copied and released viral genome would remain incomplete, lacking DR_L_-PAC1 and DR_R_-PAC2 and would require further processing before full reactivation could occur.

### 3.2. T-Loop Formation and Excision of the ciHHV-6 Genome

**Figure 2:**
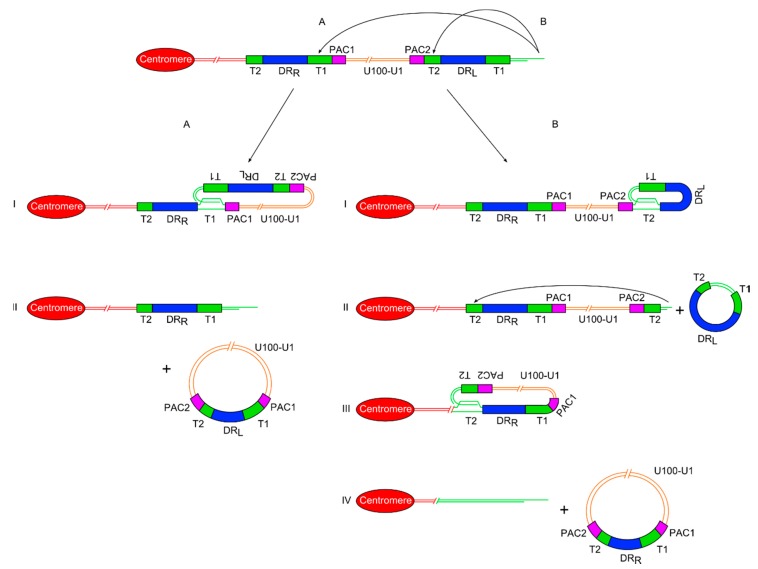
Diagram showing two proposed models of release of a ciHHV-6 genome from the telomere via t-loop processing mechanisms. (**A**) On the left side is the single t-loop model. (I) A t-loop forms between DR_L_-T1 and DR_R_-T1. (II) This t-loop facilitates release of the HHV-6 genome as a circular double stranded DNA molecule including the unique region and a recombinant DR, with restored PAC1 and PAC2 sequences. It also shows that a single DR lacking PAC sequences is retained in the telomere. (**B**) On the right side is the double t-loop model that releases the ciHHV-6 genome in two steps. (I-II) First with release of a circular DR region lacking PAC1 and 2 after t-loop formation between DR_L_-T1 and DR_L_-T2. (III-IV) This is followed by formation of a second t-loop between DR_L_-T2 and DR_R_-T2 which leads to the release of the HHV-6 genome including the unique region and a recombinant DR. In this double t-loop model, no residual DR sequences are left in the telomere.

The G-rich 3’ overhang at the end of the telomere, in association with various proteins, is capable of undergoing strand invasion into the upstream duplex DNA of the telomere. This results in the formation of a D-loop, where the overhang displaces the G-rich strand, and forms a double-stranded t-loop of telomere repeats [73]. Regulator of telomere elongation helicase 1 (RTEL1) has been identified as the helicase which preferentially resolves this structure. It is recruited by TRF2 allowing dissolution of t-loops ahead of the replisome during S phase [74]. RecQ helicases Werner syndrome RecQ-like helicase (WRN) [75] and Bloom syndrome RecQ-like helicase (BLM) [76] have also been implicated in t-loop dissolution. In the absence of sufficient RTEL1 helicase for resolution, t-loops are excised by the structure specific endonuclease complex (SLX1-SLX4) nuclease complex [77], potentially leading to substantial telomere shortening. Recent studies [24,78] have supported a model of ciHHV-6 genome release through the formation of t-loops within the ciHHV-6 genome. There are two routes to achieve this, the first is a two-stage process requiring the formation of two independent t-loops within the ciHHV-6 genome and the second just one t-loop (Figure 2). 

#### 3.2.1. Double T-Loop Formation and Excision

In the double t-loop model, the first step is single-strand invasion by the 3’ telomere overhang into the pure array of (TTAGGG)_n_ repeats at DR_L_-T2. The t-loop containing the majority of DR_L_ could then be excised as a double-stranded circular molecule by the normal telomere processing described above. Detection of severely truncated telomeres at DR_L_-T2 in lymphoblastoid cell lines **[24]** and DNA from white blood cells implies that t-loop formation can occur here. In addition extra-chromosomal circular DNA comprising DR sequences has been detected in lymphoblastoid cell lines from ciHHV-6 carriers [24] and in a T–cell line infected with HHV-6 [78]. Recently, we have found that following truncation, the telomere at DR_L_-T2 can be lengthened suggesting that some truncations occur in a stem or pluripotent cells with active telomerase. Lengthening of the truncated telomere may allow the cell to avoid the M1 checkpoint and senescence. However, if the telomere is not lengthened, a second t-loop may form between the newly formed short telomere at DR_L_-T2 and the internal DR_R_-T2. The product of a second excision event, following t-loop formation at DR_R_-T2, would be a circular viral genome with a full length unique region and a recombinant DR including a T1, T2, PAC1 and PAC2. Recombinant DR molecules have been detected at a low abundance in lymphoblastoid cell lines from ciHHV-6 carriers [24] supporting the t-loop models of HHV-6 genome release (unique region + recombinant DR). The reciprocal product from release of the circular HHV-6 genome would be a truncated telomere at the end of the chromosome but lacking any identifiable remnants of the HHV-6 genome (Figure 2).

#### 3.2.2. Single T-Loop Formation and Excision

An alternative model proposes the formation a single t-loop between the telomere and DR_R_-T1, with recombination within the T1 (or DR region) followed by excision to produce an extra-chromosomal circular DNA molecule with the entire viral genome including a single intact DR (Figure 2). As stated above, recombinant DR molecules can be detected at a low abundance in lymphoblastoid cell lines from ciHHV-6 carriers, supporting the single or double t-loop models with recombination to release the viral genome with a single intact DR. The reciprocal product in the single excision model would be a severely truncated telomere containing a residual DR without PAC1 or PAC2. As stated, individuals who are carriers of only an integrated DR have been identified during population screens but at a lower frequency than inherited ciHHV-6 carriers with a full viral genome [24,25]. Carriers of inherited ciHHV-6-DR only could arise from (i) partial HHV-6 genome insertion by homology-dependent recombination at the time of telomeric integration in the germline; (ii) HHV-6 genome excision from the telomere (as described above) in the germline giving rise to gametes and subsequently offspring with inherited ciHHV-6-DR; (iii) excision of the HHV-6 genome (as described) pre or postnatally giving rise to carriers mosaic for full inherited ciHHV-6 and ciHHV-6 DR. Further population screening and copy number analysis of DR and unique regions in a range of tissues from inherited ciHHV-6 carriers will help elucidate the underlying processes.

**Figure 3:**
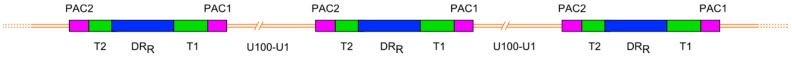
Diagram showing concatemers that would rise form rolling circle replication of a HHV-6 genome that had been excised from a telomere following t-loop formation.

Both models of t-loop formation and HHV-6 genome excision would need to be followed by rolling circle replication of the extra-chromosomal circle to form concatemers of the unique region and a DR (Figure 3). Theoretically, the concatemers could be processed through PAC1 and PAC2 to generate the packaged viral particles. 

### 3.3. ciHHV-6, Replisome Stalling, Fork Collapse and Remodelling

**Figure 4:**
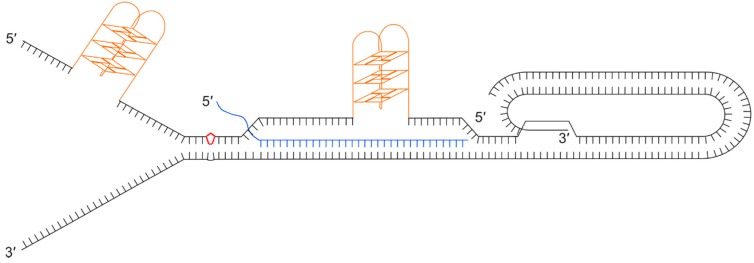
Illustration showing a collection of secondary structures that could impede progression of the replication fork through a telomere. Black lines show the double stranded telomeric DNA with a terminal t-loop and D-loop, as a result of the 3’ single stranded overhang invading the upstream duplex DNA. Orange structures show G-quadruplexes on the (TTAGGG)_n_ strand that could form during passage of the replication fork or when the G-rich strand is displaced. The diagram also illustrates the formation of a DNA:RNA hybrid between the telomeric C-rich strand and a telomere repeat-containing RNA (TERRA) molecule (blue). The red kite shape shows damaged bases, for example a pyrimidine dimer.

Any obstacles to the replisome can affect replication timing and cell cycle progression, and this can result in replication fork-stalling or collapse followed by remodelling leading to complex, multi-stranded DNA structures and double-strand breaks [79]. Currently, most evidence indicates that replication of each telomere begins at an origin of replication upstream of the telomeric DNA and travels uni-directionally through the telomere-repeat array toward the terminus and single strand overhang [80,81,82]. Consequently, a stalled replication fork within the telomeric DNA must be restarted to avoid fork-stall and formation of a double-strand break that, in the absence of a telomere maintenance mechanism, can result in sudden truncation of telomeric DNA. It is therefore important that impediments to telomere replication are removed efficiently to avoid the risk of incomplete telomere replication and stochastic formation of shortened telomeres in daughter cells. Several intrinsic properties of telomeres have been shown to be potentially problematic for their replication (Figure 4).

Given the G-rich nature of the telomere, G-quadruplexes (G4) can readily form on the lagging strand template in front of the replication machinery where the DNA duplex has been unwound. These secondary structures are known to be mutagenic and they form considerable barriers to progression of the replisome that can lead to fork-stalling, breakage and telomere loss. Telomeric protein POT1 and replication protein A (RPA) [83] inhibit the formation of G4 secondary structures by binding to the single stranded DNA. Several helicases have been shown to resolve G4s, including the 3’-5’ directed RecQ helicases, BLM [84] and WRN [85] (which may be recruited to telomeres by TRF2 and TRF1 respectively), and RTEL1, a 5’–3’ helicase [77]. t-loops may help prevent the end of the telomere being misidentified as a double-stranded break while excision of t-loops as t-circles has been proposed as a mechanism of telomere trimming [86] but if not excised or resolved they prove a considerable obstacle for the replication fork. TERRA transcripts are also believed to hinder telomere replication by forming RNA:DNA hybrids. Finally, lesion-producing DNA damage may also be more prevalent at telomeres due the nature of the DNA sequence. For example, telomeric DNA is rich in TT and CC dinucleotides, the precursors of pyrimidine dimers, and it is rich in guanine, the nucleotide that is most often oxidised [79]. To add to all these potential impediments to replication, the presence of the ciHHV-6 genome could also have a deleterious impact.

It is worth considering that truncations observed at DR_L_-T2 and excision of the entire ciHHV-6 genome could also be attributed to replication fork collapse at DR_L_-T2 and DR_R_-T2 respectively. The presence of telomere-like repeats in the ciHHV-6 genome may lead to some of the replication problems discussed above. The T1 and T2 regions may show partial binding of TRF1 and TRF2, and low levels of POT1 when single stranded. Consequently, if the RecQ helicases, WRN and BLM, are not recruited efficiently, G4 structures may be more likely to form within the ciHHV-6 genome during replication. It is conceivable therefore that replication forks are prone to stalling at T1 and T2 regions of the DRs. A viral genome excised as a result of replication fork stalling would need further processing to produce an intact DR.

## 4. Conclusions and Future Research Perspectives

In conclusion, release of the ciHHV-6A or ciHHV-6B genome from a telomere could be as a linear or a circular molecule. Such events are likely to be stochastic across a population of cells but will probably have a deleterious impact on the telomere and the cell. If excision of the viral genome occurs in stem cells, the germline or other cells that express telomerase, the suddenly truncated telomere could be rescued, facilitating survival and continued growth of the cell with the free viral genome. Assuming release of the viral genome is the first step towards full reactivation, a complete DR must be reconstituted and so excision of the ciHHV-6 genome as the double stranded circular viral genome by single or double t-loop driven mechanisms are the favoured models. The next steps that might allow progression to expression of HHV-6 genes and replication of the circular molecule are unknown but will presumably be influenced by the epigenetic state of the excised viral genome. We have proposed models for release of the ciHHV-6 genome from the telomere but many important questions remain unanswered and these must be addressed in order to understand the life cycle of HHV-6.

While there are associations between ciHHV-6 and human health, the potential link between immunocompromised individuals, HHV-6 reactivation, and disease should also be monitored. Ideally, this would be through large, multi-year studies which could accurately assess the risk between tissue transplant and disease, allowing due diligence to be exercised when transplanting ciHHV-6+ tissues into ciHHV-6- patients. Finally, large-scale aberrations of ciHHV-6 should be documented in order to established whether they could be causative in some unusual cancers, such as the HHV-8-unrelated PEL-like lymphoma case described above or in the patient with a diffuse large B-cell lymphoma displaying a marker chromosome with ciHHV-6 [87]. In the future, it may be prudent to screen individuals with these rare cancers for inherited ciHHV-6.
